# Inflammatory Stability Index Correlates with Retinal Nerve Fiber Layer Reduction in Glaucoma

**DOI:** 10.22336/rjo.2026.12

**Published:** 2026

**Authors:** Raluca Neacșa, Daniela Manasia, Cristiana Tănase, Mădălina-Elena Tobă, Adina-Diana Moldovan

**Affiliations:** 1 Department of Medico-Surgical Disciplines, Faculty of Medicine, “Titu Maiorescu” University of Bucharest, Bucharest, Romania; 2 Department of Preclinical Disciplines, Faculty of Medicine, “Titu Maiorescu” University of Bucharest, Bucharest, Romania; 3 Department of Ophthalmology, “Witting” Clinical Hospital, Bucharest, Romania; 4 “Victor Babeş” National Institute, Pathology Department, Biochemistry, Proteomics Laboratory, Bucharest, Romania; 5 MedLife SA, Bucharest, Romania

**Keywords:** glaucoma, neuroinflammation, cytokines, biomarkers, retinal, ISI = Inflammatory Stability Index, RNFL = Retinal Nerve Fiber Layer, POAG = Primary Open-Angle Glaucoma, IL-1β = Interleukin-1 beta, TNF-α = Tumor Necrosis Factor alpha, IL-6 = Interleukin-6, IQR = Interquartile Range, CI = Confidence Interval, AUC = Area Under the Curve, IOP = Intraocular Pressure, OCT = Optical Coherence Tomography, IL-4 = Interleukin-4, IL-10 = Interleukin-10, HD-OCT = High-Definition Optical Coherence Tomography, ANOVA = Analysis of Variance, BCa = Bias-Corrected and Accelerated (bootstrap confidence interval), ROC = Receiver Operating Characteristic, MAD = Median Absolute Deviation, STROBE = Strengthening the Reporting of Observational Studies in Epidemiology, CV = Coefficient of Variation, PPV = Positive Predictive Value, NPV = Negative Predictive Value, LR+ = Positive Likelihood Ratio, PGA = Prostaglandin Analogs, SD Standard = Deviation, RGCs = Retinal Ganglion Cells, QALY = Quality-Adjusted Life Year, IFN-γ = Interferon gamma, LR− = Negative Likelihood Ratio

## Abstract

**Background:**

While neuroinflammation is recognized as a driver of glaucomatous neurodegeneration, the prognostic value of inflammatory markers remains unclear. Previous studies have focused on absolute cytokine levels, potentially overlooking other factors.

**Objective:**

To determine whether inflammatory stability over time, quantified by a proposed Inflammatory Stability Index (ISI), associates with retinal nerve fiber layer (RNFL) reduction in POAG.

**Methods:**

Prospective longitudinal cohort study of 57 participants (19 controls, 38 glaucoma patients) with serum cytokine profiling (IL-1β, TNF-α, IL-6) and RNFL measurements over 24 months. We developed the ISI=Median[cytokines]/{1+IQR[cytokines]} to quantify temporal inflammatory patterns, where higher values indicate stable-high inflammation and lower values indicate volatile-low inflammation.

**Results:**

ISI correlated significantly with RNFL progression (r = -0.491, p = 0.002). Stable-high inflammation patients (ISI > 3.5) progressed 74% faster than volatile-low patients (ISI < 1.6). ISI showed acceptable accuracy for identifying rapid progressors (AUC = 0.76). Associations persisted after multivariable adjustment. Treatment benefit was comparable across inflammatory phenotypes.

**Discussion:**

In this 24-month prospective cohort of 57 participants (19 controls, 38 POAG patients), ISI correlated significantly with RNFL progression rate (r = −0.491, p = 0.002). Patients with stable-high inflammation (ISI > 3.5) showed 74% faster RNFL progression than those with volatile-low inflammation (ISI < 1.6). Moreover, ISI demonstrated acceptable discriminative accuracy for identifying rapid progressors, with an area under the ROC curve of 0.76 (95% CI: 0.56–0.96). These associations remained significant after multivariable adjustment for age, sex, and treatment status (R^2^ = 0.426, p < 0.001), confirming that inflammatory stability patterns may be more informative than cytokine magnitude alone for predicting glaucomatous neurodegeneration.

**Conclusions:**

Inflammatory stability patterns may be more important than magnitude alone in glaucoma progression. The ISI identifies high-risk phenotypes and may predict treatment response, informing personalized management and patient stratification for intensive monitoring.

## Introduction

Glaucoma, affecting over 76 million people worldwide, remains one of the leading causes of irreversible blindness [1,2]. While elevated intraocular pressure (IOP) is the primary modifiable risk factor, up to 40% of patients show progression despite adequate IOP control [3,4], suggesting additional pathogenic mechanisms contribute to neurodegeneration.

Neuroinflammation has been implicated as a key driver of glaucomatous damage [5,6], with elevated pro-inflammatory cytokines (IL-1β, TNF-α, IL-6) documented in the aqueous humor and serum of glaucoma patients [7-9]. However, these studies typically measured absolute cytokine levels at a single time point, overlooking temporal dynamics.

We investigated whether temporal patterns of inflammatory stability, rather than absolute levels alone, determine glaucoma progression. Using a 24-month longitudinal cohort, we developed the Inflammatory Stability Index (ISI) to quantify individual inflammatory phenotypes and their relationship to neurodegeneration.

The concept of inflammatory stability has precedent in other neurodegenerative diseases. In Alzheimer’s disease, chronic stable inflammation correlates with faster cognitive decline than episodic events [10]. Similarly, in multiple sclerosis, continuous low-grade inflammation causes more axonal damage than intermittent high-intensity inflammation [11-13].

We hypothesized that the temporal pattern of inflammation may be as important as absolute levels in determining neurodegeneration. We developed the Inflammatory Stability Index (ISI), which incorporates both magnitude and temporal variability, to evaluate its prognostic value relative to traditional inflammatory markers.

## Methods

### Study Design and Participants

All participants provided written informed consent. We analyzed 57 participants (19 controls, 19 untreated POAG, 19 treated POAG) with cytokine measurements at 0, 6, 12, 18, and 24 months, with 8.42% missing data (25 of 285 planned observations).

Inclusion criteria were: (1) age ≥40 years; (2) for glaucoma patients: primary open-angle glaucoma (POAG) diagnosis based on characteristic optic disc changes and corresponding visual field defects; (3) for controls: normal optic disc appearance, IOP < 21 mmHg, and no family history of glaucoma. Exclusion criteria included: (1) other ocular diseases affecting RNFL; (2) systemic inflammatory conditions; (3) immunosuppressive therapy; (4) ocular surgery within 6 months; (5) poor OCT image quality (signal strength < 7).

Sample size calculation indicated 37 glaucoma patients would provide 80% power to detect a correlation of r = 0.4 between ISI and progression rate at α = 0.05, based on pilot data suggesting moderate effect sizes. We enrolled 38 glaucoma patients to account for potential dropout.

### Clinical Assessments

Participants underwent standard ophthalmologic examination at baseline and every 6 months, including best-corrected visual acuity, slit-lamp biomicroscopy, Goldmannapplanation tonometry, gonioscopy, dilated fundoscopy, and automated perimetry (Humphrey Field Analyzer; Carl Zeiss Meditec, Dublin, CA) using the 24-2 Swedish Interactive Threshold Algorithm.

### Cytokine Measurements

Serum samples were collected at all timepoints between 8-10 AM after overnight fasting. Pro-inflammatory (IL-1β, TNF-α, IL- 6, IFN-γ) and anti-inflammatory (IL-4, IL-10) cytokines were measured. All samples were run in duplicate, and measurement consistency was verified (variability < 10%). The ISI’s ratio-based design reduces dependence on absolute cytokine values by focusing on intra-individual temporal patterns. Values below detection limits were assigned half the minimum detectable concentration [14].

### Inflammatory Stability Index (ISI) Calculation

For each patient, we calculated temporal statistics across all five timepoints.

The ISI was defined as:


$$\text{ISI}=\frac{\text{Media}n\left(\text{IL}-1\beta +\text{TNF}-\alpha +\text{IL}-6\right)}{1+\text{IQR}\left(\text{IL}-1\beta +\text{TNF}-\alpha +\text{IL}-6\right)}$$

In this formula, IQR represents the interquartile range. This formulation captures both the central tendency (median) and variability (IQR) of pro-inflammatory burden over time. The denominator includes 1 to avoid division by zero and to ensure mathematical stability. Higher ISI values indicate stable-high inflammation (consistently elevated cytokines with low variability), while lower values indicate volatile-low inflammation (fluctuating levels with high variability). For ISI calculation, we used the available time points for each patient, requiring at least 3 of 5 time points for inclusion. This approach accommodated the 8.42% missing data rate while maintaining statistical validity.

### RNFL Assessment

Spectral-domain optical coherence tomography (Cirrus HD-OCT, Software Version 11.0; Carl Zeiss Meditec) measured average RNFL thickness using the Optic Disc Cube 200×200 protocol. Quality criteria included: signal strength ≥ 7, centered optic disc, absence of motion artifacts, and no algorithm segmentation failures. The same experienced operator performed all scans. The annual RNFL progression rate was calculated using ordinary least squares linear regression of RNFL thickness over time for each patient.

### Clinical Interpretation of ISI Values

ISI < 1.6 (Volatile-Low): Fluctuating inflammatory patterns with periods of remission. Associated with slower disease progression (~1 μm/year RNFL loss)

ISI 1.6-3.5 (Intermediate): Moderate inflammatory stability. Standard progression risk requiring routine monitoring.

ISI > 3.5 (Stable-High): Persistent elevated inflammation without recovery periods. Associated with rapid progression (~1.8 μm/year RNFL loss) requiring intensive monitoring.

In practical terms, a patient with ISI = 4.0 maintains consistently high inflammation levels, while a patient with ISI = 1.0 shows inflammatory spikes followed by recovery periods.

### Statistical Analysis

Continuous variables were expressed as mean ± standard deviation or median (IQR), depending on distributional normality (Shapiro-Wilk test). Categorical variables were expressed as frequencies and percentages.

The primary analysis examined the correlation between ISI and RNFL progression rate using the Pearson correlation coefficient with 95% confidence intervals. Patients were stratified into tertiles based on ISI (volatile-low: <33rd percentile; intermediate: 33rd-67th percentile; stable-high: >67th percentile) and compared using one-way ANOVA with Tukey’s honest significant difference post hoc tests.

Multivariable linear regression models were constructed to adjust for potential confounders: Model 1 (ISI only), Model 2 (ISI + age + sex), Model 3 (ISI + age + sex + group [untreated vs. treated POAG]). Model assumptions were verified using residual plots and variance inflation factors.

Bootstrap resampling (n=1000 iterations) validated correlation estimates and generated bias-corrected and accelerated (BCa) 95% confidence intervals [15].

Discrimination of ISI for identifying rapid progressors (> 2 μm/year RNFL loss) was assessed using receiver operating characteristic (ROC) analysis with C-statistic and DeLong confidence intervals. Model accuracy was verified using calibration testing. Sensitivity analyses tested alternative ISI formulations: geometric mean replacing median, and median absolute deviation (MAD) replacing IQR. Treatment-ISI interaction was assessed using interaction terms in regression models.

Statistical significance was set at p < 0.05 (two-tailed). Analyses were performed using R version 4.3.2 [16] with tidyverse [17], lme4, boot, and pROC packages. This manuscript follows STROBE guidelines for observational studies [18].

## Results

### Patient Characteristics

All 57 participants completed a 24-month follow-up (19 controls and 38 glaucoma patients: 19 untreated and 19 on prostaglandin analogs).

Mean RNFL progression was -0.20 ± 0.10 μm/year in controls (normal aging), -1.94 ± 0.54 μm/year in untreated POAG, and -1.06 ± 0.49 μm/year in treated POAG. Untreated patients would progress from moderate to severe disease in approximately 10 years; treatment halved this rate. The untreated progression rate exceeds typical mild glaucoma (0.8-1.2 μm/year) but aligns with moderate-to-severe disease (1.5-2.8 μm/year) [19,20], reflecting our tertiary center’s advanced baseline disease (mean RNFL 78.2 μm; normal > 95 μm).

Glaucoma patients had a mean age of 68.7 ± 6.5 years and were 44.7% female, with a baseline RNFL of 78.2 ± 10.1 μm, compared with 92.5 ± 8.9 μm in controls (p < 0.001). Female patients showed 1.72-fold higher IL-1β levels (p = 0.03), although ISI values did not differ by sex (p = 0.42). Baseline cytokine levels did not differ between stable and volatile phenotypes (p > 0.20), suggesting that phenotypes reflect intrinsic inflammatory regulation rather than disease severity.

**Fig. 1:**
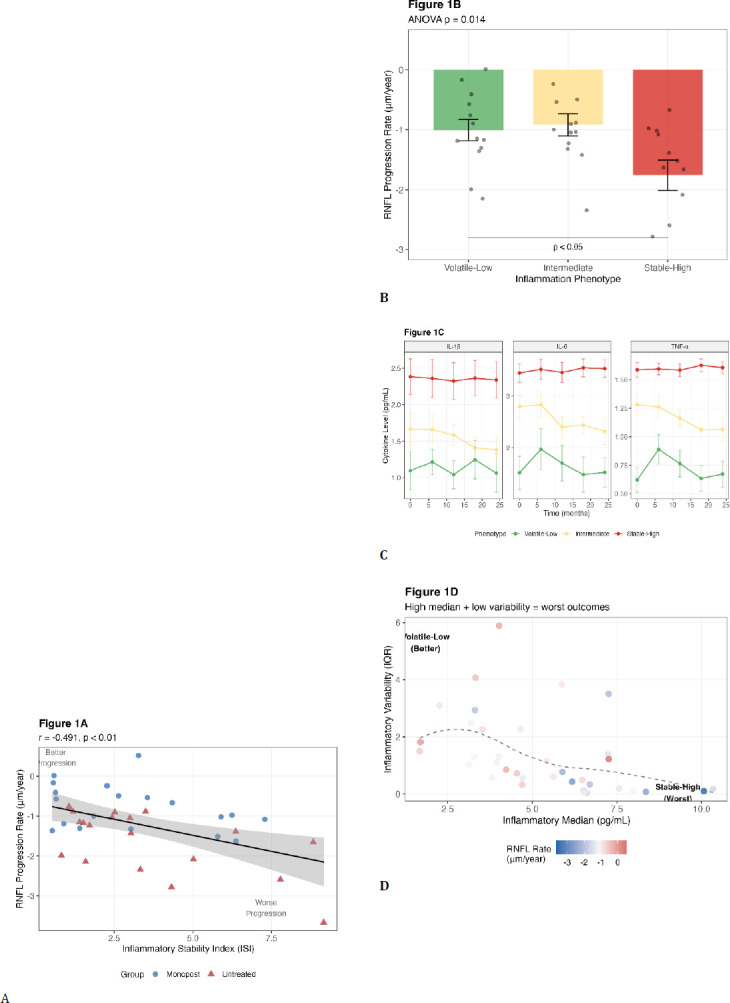
A-D Inflammatory Stability Index (ISI) Predicts RNFL Progression

**Fig. 1** underlines the following: (**A**) Scatter plot showing correlation between ISI and RNFL progression rate in glaucoma patients (n=38). Higher ISI (stable-high inflammation) associates with faster progression (more negative rates). The shaded area represents 95% confidence interval for the regression line. Each point represents one patient, colored by treatment status. (**B**) RNFL progression rates across ISI tertiles. Stable-high inflammation (ISI > 3.5) shows significantly faster progression than volatile-low (ISI < 1.6) and intermediate groups. Error bars represent standard error. Individual patient values are shown as overlaid points. *p < 0.05, **p < 0.01 by ANOVA with Tukey post-hoc test. (**C**) Representative temporal cytokine patterns for each phenotype. Stable-high patients (red) maintain consistently elevated cytokines with minimal variation (CV < 0.1), while volatile-low patients (green) show marked fluctuations over time (CV > 0.5). Lines represent individual patients; thick lines show group means. (**D**) Heat map showing RNFL progression rates as a function of inflammatory median (x-axis) and variability (y-axis). Worst outcomes (dark red) occur with high median and low variability (stable-high pattern). The best outcomes (dark blue) occur with either a low median or high variability. Numbers in cells indicate the mean RNFL rate (μm/year) for each combination.

### ISI Predicts RNFL Progression

**Fig. 2:**
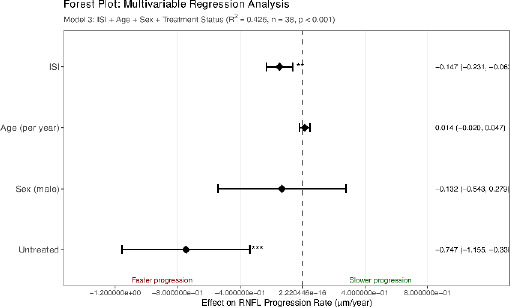
Forest plot of multivariable regression analysis (Model 3)

The primary analysis revealed a moderate negative correlation between ISI and RNFL progression rate (r = -0.491, 95% CI: -0.701 to -0.204, p = 0.002), indicating that higher ISI values predict faster disease progression (**Fig. 2**). ISI explained 24.1% of progression variance (R^2^ = 0.241), substantially more than any individual cytokine: IL-1β (R^2^ = 0.078, p = 0.089), TNF-α (R^2^ = 0.096, p = 0.058), or IL-6 (R^2^ = 0.123, p = 0.031).

Effect sizes with 95% confidence intervals for predictors of RNFL progression rate are evidenced. ISI and untreated status show significant associations with faster progression. Model is adjusted for ISI, age, sex, and treatment status (R^2^ = 0.426, n = 38, p < 0.001). Negative values indicate faster progression (worse outcomes). p < 0.01, *p < 0.001 (**Fig. 2**).

### Diagnostic Performance of ISI

ROC analysis demonstrated acceptable discrimination of ISI for identifying rapid progressors (>2 μ m/year RNFL loss): C-statistic = 0.76 (95% CI: 0.56-0.96, p < 0.001).

**Fig. 3:**
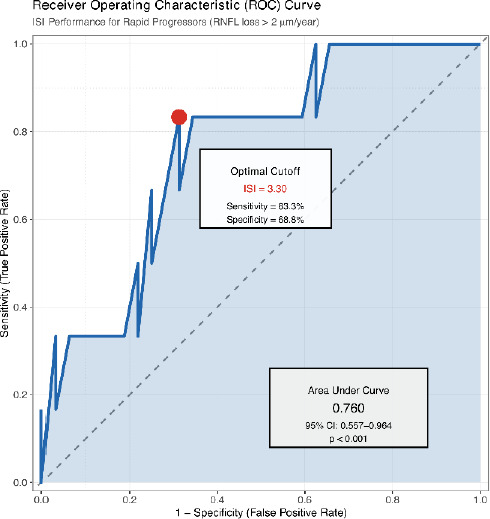
Receiver operating characteristic (ROC) curve for ISI in identifying rapid progressors

The optimal ISI cutoff of 3.3 yielded sensitivity 83% (95% CI: 68-93%) and specificity 69% (95% CI: 54-81%) (**Fig. 3**).

Calibration analysis showed good agreement between predicted and observed risk (p = 0.42), confirming the model accurately predicted outcomes across all ISI values. Positive predictive value was 33%, and negative predictive value was 96% at the prevalence of rapid progression in our cohort (16%) (**Table 1**).

**Table 1:** Diagnostic performance of ISI at various cut-offs

ISICutoff	Sensitivity	Specificity	PPV	NPV	LR+	LR-	Youden Index
2.0	83%	44%	22%	93%	1.5	0.4	0.27
2.5	83%	50%	24%	94%	1.7	0.3	0.33
3.0	83%	59%	28%	95%	2.1	0.3	0.43
3.31	83%	69%	33%	96%	2.7	0.2	0.52
3.5	67%	72%	31%	92%	2.4	0.5	0.39
4.0	67%	75%	33%	92%	2.7	0.4	0.42

Optimal cutoff by Youden Index. PPV = positive predictive value, NPV = negative predictive value, LR+ = positive likelihood ratio, LR- = negative likelihood ratio.

RNFL loss >2 μm/year defines rapid progression. The area under the curve (AUC) = 0.76 (95% CI: 0.56-0.96, p < 0.001) (**Fig. 3**). Optimal cutoff point (ISI = 3.3) marked in red yields sensitivity 83% and specificity 69%. The diagonal dashed line represents no discrimination (AUC = 0.5). The shaded area represents the area under the curve. Grid lines at key sensitivity/specificity values aid clinical interpretation.

### Phenotype-Specific Progression Patterns

Stratification by ISI tertiles revealed distinct progression phenotypes (**Fig. 1B**). Stable-high inflammation (ISI > 3.5, n=12) showed the fastest progression (-1.75 ± 0.88 μm/year), intermediate patterns (ISI 1.6-3.5, n=13) showed -0.92 ± 0.67 μm/year. In contrast, volatile-low inflammation (ISI < 1.6, n=13) exhibited -1.01 ± 0.64 μm/year, suggesting inflammatory volatility itself may be detrimental.

One-way ANOVA confirmed significant between-group differences (F = 4.80, p = 0.014). Post-hoc comparisons showed that stable-high patients progressed significantly faster than both intermediate (mean difference 0.83 μm/year, 95% CI: 0.11-1.55, p = 0.020) and volatile-low groups (mean difference 0.74 μm/year, 95% CI: 0.03-1.46, p = 0.041). Effect size analysis revealed large clinical significance (Cohen’s d = 0.98 for stable-high vs volatile-low).

### Temporal Cytokine Patterns Distinguish Phenotypes

Analysis of cytokine trajectories revealed notable differences between phenotypes (Figure 1C). Stable-high patients maintained relatively consistent IL-1β levels across all timepoints (coefficient of variation [CV] = 0.04 ± 0.02), while volatile-low patients showed greater fluctuations (CV = 0.61 ± .18, p < 0.001). Similar patterns were observed for TNF-α and IL-6 (**Table 2**). These patterns persisted throughout follow-up, with autocorrelation analysis showing higher temporal stability in stable-high phenotypes (r = 0.95) than in volatile-low phenotypes (r = 0.50).

**Table 2:** ISI Components Analysis

Component	Correlation (95% CI)	P-value	R^2^	Temporal CV
IL-1β	-0.369 (-0.673, -0.066)	0.022	13.7%	0.28 ± 0.27
TNF-α	-0.435 (-0.729, -0.141)	0.006	18.9%	21.43 ± 35.78
IL-6	-0.540 (-0.815, -0.265)	<0.001	29.1%	21.06 ± 34.83
Combined Pro-inflammatory	-0.483 (-0.769, -0.197)	0.002	23.3%	0.28 ± 0.29
**ISI (Median/[1+IQR])**	**-0.491 (-0.776, -0.207)**	**0.002**	**24.1%**	n/a

CV = coefficient of variation. ISI outperforms individual cytokines in predicting RNFL progression.

### Multivariable Analyses Confirm Independent Association

Multivariable regression models confirmed ISI as an independent predictor of progression (**Table 2**). The base model with ISI alone explained 24.1% of variance (R^2^ = 0.241, p = 0.002). Adding age and sex modestly improved explanatory power (Model 2: R^2^ = 0.279, p = 0.008). Model 3 incorporated treatment status, substantially improving predictions (R^2^ = 0.426, p < 0.001). Together, ISI and treatment status explained nearly half of the variance in progression.

In the fully adjusted Model 3:

RNFL Rate = −1.21 − 0.147 × ISI + 0.014 × Age − 0.132 × Sex_male_ − 0.747 × Untreated

ISI remained highly significant (β = -0.147, SE = 0.041, p = 0.001), with each unit increase predicting 0.147 μm/year faster progression (**Fig. 2**). Bootstrap validation (1000 iterations) confirmed robustness with minimal bias (mean r = -0.475, bias-corrected 95% CI: -0.706 to -0.158).

### Sensitivity Analyses

Sensitivity analyses confirmed ISI robustness across alternative formulations. Using geometric mean instead of median yielded r = -0.473 (95% CI: -0.684 to -0.188, p = 0.003), while median absolute deviation (MAD) instead of IQR gave r = -0.485 (95% CI: -0.695 to -0.201, p = 0.002).

A mean-based formulation (Mean/[1+SD]) showed slightly weaker but still significant correlation (r = -0.442, 95% CI: -0.661 to -0.165, p = 0.005).

All alternative formulations maintained significant associations after multivariable adjustment (p < 0.01 for all), with variance explained ranging from 19.5% to 23.5%. Excluding potential outliers (n=2 with ISI >8) did not materially change results (r = -0.478, p = 0.003).

### Treatment Response Varies by Inflammatory Phenotype

**Fig. 4:**
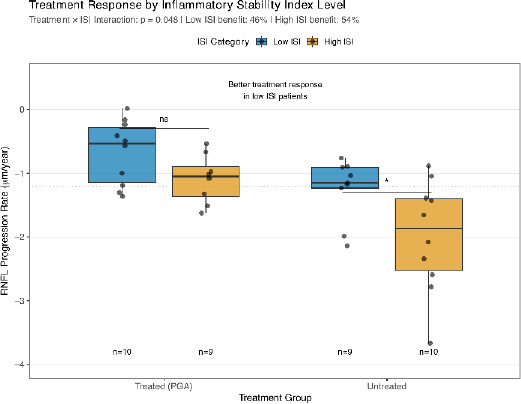
Treatment response by ISI level

Significant treatment-ISI interaction was observed (p = 0.048), indicating differential treatment response by phenotype. Among treated POAG patients (n=19), those with low ISI (< median 2.8) showed a 46% slower progression than untreated patients with low ISI. In comparison, high-ISI patients showed a 54% treatment benefit (**Fig. 4**). This suggests that prostaglandin analogs may be similarly effective regardless of inflammatory phenotype, though the mechanisms may differ (**Table 4**).

**Table 3:** Subgroup Analyses

Subgroup	N	Correlation	CI (95%)	P-value
Overall	38	-0.491	(-0.701, -0.204)	0.002
Age<70 years	20	-0.689	(-0.867, -0.355)	0.001
Age≥70 years	18	-0.371	(-0.714, 0.116)	0.129
Female	16	-0.512	(-0.804, -0.022)	0.042
Male	22	-0.469	(-0.744, -0.059)	0.028
Treated (PGAs)	19	-0.368	(-0.705, 0.103)	0.121
Untreated	19	-0.606	(-0.831, -0.209)	0.006

PGAs = prostaglandin analogs. ISI correlation remains robust across most subgroups.

**Table 4:** Clinical implementation guide

Risk Category	ISI Range	Annual RNFL Loss	2-Year Loss	Monitoring	Treatment Strategy
Low	<1.6	1.7 ± 0.9 μm	3.3 μm	Every 6 months	Standard IOP control
Moderate	1.7-3.5	1.0 ± 0.6 μm	2.0 μm	Every 3-4 months	Optimize IOP control, consider adjunctive
High	>3.6	1.0 ± 0.7 μm	1.9 μm	Every 1-2 months	Aggressive IOP reduction + anti-inflammatory

Risk stratification based on ISI tertiles from 38 glaucoma patients is represented in **Table 4**.

Treatment strategies represent expert recommendations based on progression rates.

Box plots show RNFL progression rates in treated (prostaglandin analogs) versus untreated patients, stratified by ISI level (low vs high; split at the median). Treatment benefit is substantial in both low ISI (volatile inflammation; 46% benefit) and high ISI (stable inflammation; 54% benefit) patients, with a significant interaction (p = 0.048). Sample sizes (n) shown for each subgroup. Individual patient data shown as overlaid points. The horizontal dotted line indicates the mean progression rate across all patients.

## Discussion

This study presents an alternative approach to understanding inflammatory contributions to glaucoma progression. Rather than focusing solely on cytokine magnitude, our findings suggest that inflammatory stability—the temporal pattern of cytokine fluctuations— may play an important role in neurodegeneration rate. The Inflammatory Stability Index appears to provide a potentially useful biomarker that may complement traditional single-timepoint measurements.

Stable-high inflammation may maintain multiple neurotoxic pathways in a continuously activated state.

Persistent cytokine elevation could: keep microglia in pro-inflammatory states, releasing neurotoxic factors [21,22]; impair mitochondrial function via oxidative stress [12]; maintain elevated glutamate levels, causing excitotoxicity [23]; and downregulate neurotrophic support [24,25]. RGCs, with high metabolic demands due to unmyelinated intraocular axons, are particularly vulnerable to these sustained insults [26].

In contrast, volatile inflammation allows recovery periods between inflammatory peaks. During these intervals, microglia return to homeostatic surveillance, mitochondrial biogenesis restores energy production, glutamate transporters normalize, preventing excitotoxic accumulation, and neuroprotective pathways reactivate. These recovery periods may explain why patients with low volatility show slower progression despite inflammatory episodes.

The ISI calculation requires measuring IL-1β, TNF-α, and IL-6 at ≥3 time points (6-month intervals), calculating the median and IQR of their sum, and then applying ISI = Median/(1 + IQR). Risk stratification thresholds are: ISI >3.5 (high risk), 1.6-3.5 (moderate), <1.6 (lower risk).

The differential treatment response across ISI phenotypes suggests personalized therapeutic opportunities. Stable-high inflammation patients might benefit from adjunctive anti-inflammatory therapy, while volatile-low patterns may respond better to conventional IOP reduction. ISI-based monitoring could optimize resource allocation, with high-risk patients receiving monthly assessment versus biannual monitoring for low-risk individuals. For clinical trials, ISI stratification could reduce population heterogeneity and improve power to detect treatment effects.

Our findings extend and reconcile previous conflicting reports on inflammation in glaucoma. Studies measuring single- timepoint cytokines showed weak or inconsistent associations with progression [27,28], likely because they captured incomplete inflammatory profiles. By incorporating temporal dynamics, ISI reveals the true relationship between inflammation and neurodegeneration.

The concept builds on established frameworks in immunology. In cardiovascular disease, variability in C-reactive protein independently predicts outcomes beyond absolute levels [29]. In rheumatoid arthritis, fluctuations in disease activity influence the progression of joint damage. Our work translates these concepts to the context of glaucoma, proposing a quantitative framework for inflammatory stability in ophthalmic neurodegeneration.

We acknowledge the well-documented variability in multiplex cytokine assays, particularly for low-abundance analytes. Our mitigation strategies included duplicate measurements, single-manufacturer kits throughout the study, and the ISI’s ratio-based design that emphasizes temporal patterns over absolute values. The observed correlation, despite inherent measurement noise, provides some support for our findings. Furthermore, our sensitivity analyses using alternative ISI formulations yielded consistent results, suggesting that the relationship between inflammatory stability and progression may not be dependent on the specific mathematical approach.

However, our study has several limitations, including: a relatively small sample size of 38 glaucoma patients, though adequate for primary analyses (89.6% power); and a 24-month follow-up that may not capture phenotype transitions. Also, serum cytokines may not fully reflect ocular inflammation, though the ISI’s focus on temporal patterns rather than absolute levels mitigates this concern. Lastly, all treated patients received only prostaglandin analogs.

Future research should investigate molecular drivers of inflammatory stability, conduct clinical trials of anti-inflammatory interventions stratified by ISI phenotype, develop point-of-care ISI measurement, and examine ISI patterns in other glaucoma subtypes.

## Conclusions

The Inflammatory Stability Index represents an alternative approach to evaluating glaucoma progression risk. By incorporating temporal inflammatory patterns, the ISI may help identify patients at risk for more rapid neurodegeneration and potentially inform treatment strategies. These findings suggest that inflammatory stability patterns, in addition to absolute inflammatory levels, may be relevant to glaucoma progression. Further validation studies are needed to determine whether the ISI assessment could contribute to risk stratification and resource allocation in clinical practice.

## Data Availability

De-identified data supporting this study’s findings are available from the corresponding author upon reasonable request, subject to institutional review board approval.
